# Digital Divide in Awareness, Want, and Adoption Across Diverse eHealth Services: Cross-Sectional Survey of Inpatients in Jinan, China

**DOI:** 10.2196/72297

**Published:** 2025-10-30

**Authors:** Wenjie Shi, Daopeng Duan, Shiju Dong, Zexuan Yu, Jiajia Li

**Affiliations:** 1 Department of Social Medicine and Health Management, School of Public Health, Cheeloo College of Medicine, Shandong University, 44 WenHua West Road, Li'xia District, Jinan, 250012, China, 86 053188382269, 86 053188382553; 2 Binzhou Municipal Health Commission, Binzhou, China; 3 NHC Key Lab of Health Economics and Policy Research, Shandong University, Jinan, China; 4 Shandong Provincial Key New Think Tank, Center for Health Management and Policy Research, Shandong University, Jinan, China; 5 Department of Biostatistics, School of Public Health, Brown University, Providence, RI, United States

**Keywords:** eHealth, digital divide, inpatients, hospitalized patients, factors

## Abstract

**Background:**

Despite numerous research studies examining the digital divide in the context of eHealth, studies specifically targeting inpatients remain limited. In comparison with the general population, inpatients typically undergo a progression from outpatient consultations to inpatient treatment and subsequent out-of-hospital rehabilitation. This sequential process gives rise to requirements across nearly all usage scenarios of eHealth services. Therefore, this study focuses on inpatients with potential interest in eHealth as the research subjects.

**Objective:**

This study aimed to analyze the digital divide in the awareness of, want for, and adoption of diverse eHealth services and further explore factors influencing these disparities among inpatients.

**Methods:**

A cross-sectional study was conducted in 2023, involving 1322 inpatients aged ≥15 years from 3 tertiary hospitals in Jinan, China. Data were collected through a face-to-face questionnaire survey. eHealth services were categorized into 3 types (ie, information-based, treatment intermediary, and treatment eHealth services). The adoption gap ratio is used to explore the gap between eHealth services’ adoption and awareness or want. The awareness, want, and adoption gap segment matrix was used to further analyze the digital divide in eHealth services and categorize inpatients into 4 groups (ie, opened, perception deficiency, desire deficiency, and closed group), each of which was further divided into 4 subcategories (ie, strong, generic, want-bias, and awareness-bias). Binary logistic regression was used to explore potential influencing factors of awareness, want, and adoption across diverse eHealth services.

**Results:**

The results showed that of 1322 inpatients, 1204 (91.1%) have awareness of eHealth services, 1169 (88.4%) have want for eHealth services, and 847 (64.1%) have adopted 1 or more of these services. Digital divides were observed in information-based eHealth, treatment intermediary, and treatment eHealth services, with adoption gap ratios reaching 32.1%, 34.1%, and 66.5%, respectively. Notably, all 3 eHealth services fell into the opened group. Among the 3 services, information-based eHealth services were located in the want-bias subgroup, treatment services belonged to the generic subgroup, and treatment intermediary services fell into the strong group. Binary logistic regression revealed that the influence of age, place of residence, educational attainment, income, self-rated health, chronic disease, eHealth literacy, perceived usefulness, and perceived ease of use on the awareness of, want for, and adoption of eHealth services showed notable difference and differed significantly depending on the types of eHealth services.

**Conclusions:**

This study provides empirical evidence on the existence of a digital divide in awareness, want, and adoption across diverse eHealth services among inpatients in Jinan, China. Given the promise and opportunities that eHealth services increase access to health care, future digital interventions should both address or bridge the digital divide in various eHealth services and consider the implementation of differentiated marketing strategies for diverse eHealth services.

## Introduction

### Background

The World Health Organization emphasizes that digital technologies are essential components and enablers of sustainable health systems and universal health coverage [1]. Despite robust support and the rapid expansion of eHealth services in many countries [2], a significant digital divide remains [3].

Marginalized and vulnerable populations—such as older adults, individuals with limited education, low-income groups, and rural residents—face substantial challenges in accessing and using eHealth services [2,4]. These challenges include acquiring necessary internet-enabled devices, developing sufficient internet usage skills, and improving digital literacy [5,6]. Such barriers increase their risks of inadequate internet access and poor eHealth literacy, further exacerbating the digital divide they experience [7-9]. This divide in eHealth utilization between users and nonusers undermines the potential benefits of eHealth services, limiting their capacity to contribute effectively to improved health outcomes [2].

On the basis of consumer purchasing decision model or new techniques of adoption decision-making [10,11], individuals’ engagements with eHealth services follow a hierarchical progression through 3 stages: awareness, want, and adoption [12]. Adequate knowledge of emerging technologies fosters a more rational want for eHealth services [13]. For example, a study conducted in a Spanish border community revealed that eHealth literacy positively influences the intention to use telehealth services [14]. However, it is important to note that a need for eHealth does not always lead to actual adoption. As health status deteriorates and physical functionality declines, older adults often exhibit a heightened demand for eHealth services, particularly those that help overcome geographical constraints [15]. However, because of vision impairments and reduced learning capacities [16], their actual utilization of eHealth remains significantly lower than that of younger individuals [17]. Nevertheless, once older adults begin using eHealth services, they tend to show greater persistence in using health management apps compared to their younger counterparts [18].

According to their functions of eHealth services, eHealth services’ typical classifications include health information services [19], e-consulting services [20], online appointment booking [21], and eHealth commerce [22]. Health information services, which involve activities such as health information seeking, health risk assessments, and personal health records, are prevalently used globally [23-27]. E-Consulting, introduced in the early 2000s [28,29], has seen significant adoption in countries such as England and Canada [30-32], although its uptake has been slower in low- and middle-income countries [33]. Online appointment booking, including online scheduling and access to test and laboratory results [34], has been implemented in many countries, including England, Australia, and Canada [35-37]. eHealth commerce presents a promising opportunity to expand access to medications and has been implemented in many countries [38]. However, significant disparities exist in the formats and complexity of these eHealth services [33,39,40], which may result in varying levels of awareness, want, and adoption among individuals across different eHealth service categories [41-45]. Unfortunately, there is a lack of studies that specifically examine the awareness of, want for, and adoption of eHealth services within these distinct categories.

### Study Objectives

This study specifically focuses on hospitalized patients. Hospitalized patients often encounter challenges that exacerbate their exposure to a deeper digital divide and increase their demands for eHealth services [46,47]. These challenges include mobility impairments [48], prolonged waiting times [49], and additional barriers to follow-up care and chronic disease management after discharge [50-52]. The heightened demand for eHealth services among inpatients highlights their importance as a key target group for such interventions. Addressing the digital divide in eHealth services for this population is critical, as it may provide valuable insights for other countries in developing targeted strategies to promote eHealth adoption. Therefore, this study aimed to analyze the digital divide in awareness of, want for, and adoption of information-based, treatment intermediary, and treatment eHealth services using the awareness, want, and adoption (AWAG) segment matrix. In addition, it seeks to explore the underlying factors contributing to these disparities.

## Methods

### Clinical Context

In China, grade A tertiary hospitals (commonly referred to as *Sanjia* hospitals) represent the highest level of medical institutions, with responsibilities that encompass the provision of specialized health care, the advancement of medical education, and the conduct of advanced research [53]. Considering the differences in models of eHealth services, the hierarchy, and representativeness of patient sources, this study purposively selected 2 grade A tertiary hospitals and 1 tertiary hospital in Jinan, recognized for offering eHealth services, as the sampling sites. These 3 general hospitals represent distinct tiers of tertiary health care institutions, including national-level hospitals, provincial hospitals, and municipal hospitals. They serve diverse patient populations and reflect varying scales of hospital organization. Each facility is equipped with comprehensive inpatient departments and exhibits unique characteristics in the development of eHealth [54-57], as detailed in Table 1.

**Table 1.** Characteristics of 3 tertiary hospitals in Jinan for the eHealth survey from June to October 2023, including their eHealth service models and functional features.

Hospitals	Characteristic
A	Innovative “internet plus smart medical” model One-stop-shop online follow-up service Health science communication popularization matrix based on short video
B	Docking with Jinan “internet+medical health” convenience service platform Digital inpatient ward system The smart hospital built is in the forefront of Jinan municipal hospitals
C	The most functional internet hospital in the province A full range of eHealth services

### Study Design and Data Collection

In this study, the sample size calculation accounted for the issue of multiple comparisons. To address this, the significance level (*α*=.0167, 0.05/3) was adjusted using multiple Bonferroni corrections. The calculation was performed using the following standard formula, indicating that a minimum of 895 participants was needed. Considering an anticipated 30% nonresponse rate, the final required sample size was adjusted to 1279 participants.


$$n=\frac{Z_{1-\alpha /2}^{2}\times p(1-p)}{\delta^{2}}$$

where $Z_{1-\alpha /2}$= 2.393, p = 0.5, and δ= 0.04.

This study used a multistage stratified sampling approach to select participants from 3 participating hospitals. First, the sample size for each hospital was allocated proportionally based on the number of beds in each respective hospital. Subsequently, inpatients were randomly selected from all departments, excluding the emergency and obstetric departments. Specifically, in hospital A, 587 inpatients were selected from 17 departments. In hospital B, 207 inpatients were randomly chosen across 15 departments. In hospital C, 484 inpatients were selected from 15 departments. All wards within each department were included in the investigation, and a total of 305, 104, and 268 wards were included from hospitals A, B, and C, respectively. Two bed numbers were randomly selected using a random number method, and the patients occupying the corresponding beds in each ward were systematically surveyed. If the invited patient declined, the patient in the adjacent bed was approached as a replacement. As a result, a total of 1354 inpatients participated in this survey.

A face-to-face questionnaire survey was conducted across inpatient departments in these hospitals from June to October 2023. The investigators are interns majoring in preventive medicine, who have a relatively high level of medical literacy. To ensure the data quality, a comprehensive training program was implemented to clarify the questionnaire’s content and establish standardized criteria for questioning before conducting the survey. All respondents completed the questionnaire face-to-face with trained investigators, after providing their informed consent and signing the questionnaire.

This survey recruited inpatients based on the following inclusion criteria: (1) aged ≥15 years; (2) able to communicate effectively and complete questionnaires independently or with assistance; (3) not hospitalized due to childbirth or accidental injuries; (4) no history of major mental illness, language impairment, or cognitive impairment; and (5) provided informed consent. After excluding samples with missing key information or those that did not meet the inclusion criteria, a total of 1322 inpatients from 3 hospitals were included as survey participants.

### Ethical Considerations

The study was approved by the Ethics Committee of the School of Public Health, Shandong University, P.R. China (LL20230602). Participants who provided written informed consent were included in the study. Data were collected anonymously by the research team and stored securely in locked files. Participation was entirely voluntary, and no compensation was offered for participation.

### Measures

#### Measures of Dependent Variables

This study examined 12 eHealth services based on the established definition and scope of eHealth [23]. Existing literature indicates that eHealth can produce 3 primary effects.

1. The signaling effect: The internet enables patients to access and evaluate information about health care services, providers, and their quality at a reduced cost [58]. This helps mitigate information asymmetry in health care, which, in turn, enhances individuals’ health literacy, improves patient-provider matching efficiency, and elevates the overall quality of care [59-61].
2. The intermediary effect: eHealth facilitates the use of various nondiagnostic and nontreatment resources, such as online triage and appointment scheduling for examinations and surgeries. These services enhance both the accessibility and equity of high-quality medical resources by leveraging eHealth’s intermediary role [62-64].
3. The substitution effect: a range of medical services, including online consultations, telemedicine, and follow-up care, expands the allocation of health care resources through the application of information technology. By substituting traditional in-person services, eHealth improves the fairness and accessibility of high-quality medical care [65,66].

On the basis of these 3 effects and the stages of the patient journey [67], these 12 services were categorized into 3 groups: information-based services, treatment intermediary services, and treatment services, as detailed in Table 2. Information-based services provide health and medical information aimed at enhancing health literacy while reducing costs [68,69]. Treatment intermediary services support the preadmission process by leveraging nonmedical resources, thereby improving accessibility and the efficiency of health care delivery [21,68]. Treatment services, on the other hand, use information technology to optimize the allocation of medical resources, complement traditional health care practices, and promote equitable access to high-quality medical services [70-72].

**Table 2.** Classification of 12 eHealth services by functional role as information-based, treatment intermediary, and treatment services.

Items	eHealth services
Information-based services	Seek disease or health information online Seek doctor or hospital information online Seek medical review information online (patients’ evaluation of doctors) Give or receive peer-to-peer feedback about health status in online communities or chat platforms
Treatment intermediary services	Outpatient appointment booking online Pay medical bills online Access electronic medical records and medicinal examination reports online Appoint a medical examination or surgery online Online hospitalization appointment Online drugstore purchases or online pharmacy, excluding dietary supplements
Treatment services	Electronic consulting, including email, chat, or video health care consultations Chronic disease monitoring and management, online telemonitoring, or remote monitoring to manage chronic diseases, such as social networking or eHealth communities, telehealth, and mobile health, including wearable devices or apps

In this study, the awareness, want for, and adoption of eHealth services were treated as dependent variables. Inpatients were asked whether they had heard of any eHealth services (1=no, 2=yes). The awareness of eHealth services was categorized as 1 if the inpatient had heard of them and 0 otherwise. For patients who had not heard of eHealth services, the investigator provided a brief explanation of their purpose and functionality. Following this explanation, patients were asked about their intention to use eHealth services (1=no, 2=yes). Want for eHealth was then categorized as 1 if the patient expressed a willingness to use them and 0 otherwise. In addition, inpatients were asked about their experience with using eHealth services (1=no, 2=used with the help of family members, 3=used independently). Adoption of eHealth services was categorized as 1 if the inpatient reported using them independently or with the help of family members and 0 otherwise.

#### Measures of Independent Variables

On the basis of Wilson’s model of information behavior [73], this study divided the factors influencing the use of eHealth services into digital technology factors, health status, and general demographic characteristics.

#### Digital Technology Factors

eHealth literacy [60], perceived usefulness, and perceived ease of use [74] are categorized as digital technology factors.

The eHealth literacy scale (eHEALS), an 8-item instrument, was used to measure eHealth literacy [75]. Responses were collected using a 5-point Likert scale, ranging from *very inconsistent* to *very consistent*. The total score, calculated as the sum of scores across all 8 items, ranged from 8 to 40, with higher scores indicating greater eHealth literacy. eHEALS has demonstrated strong validity and reliability in China [76]. In this study, the Cronbach α for eHEALS was .976, indicating excellent internal consistency.

To measure inpatients’ acceptance of eHealth services, the constructs of perceived usefulness and perceived ease of use, derived from the Technology Acceptance Model proposed by Davis, were used [77]. Each construct was measured using 4 items, scored on a 5-point Likert scale, ranging from *very inconsistent* to *very consistent*. Total scores ranged from 4 to 20, with higher scores reflecting a more favorable evaluation of eHealth services. In this study, Cronbach α was .944 for perceived usefulness and 0.969 for perceived ease of use, indicating high reliability.

### Health Status

Health status included self-rated health (SRH) and chronic disease (no=1, yes=2). SRH was initially assessed using 5 categories: very poor, poor, fair, good, and very good. For the purposes of this study, SRH was reclassified into 3 categories: negative (very poor or poor), fair, and positive (good or very good).

### General Demographic Characteristics

General demographic characteristics also were selected in this study, such as sex (male=1, female=2), age (in years), marital status (married=1, unmarried=2), educational attainment (in years), and place of residence (urban=1, rural=2). Income, categorized into 3 groups based on per capita disposable household income and the average per capita urban and rural household income, was also considered [78]: lowest (below 40% of the per capita disposable household income of Jinan), middle (between 40% and 100% of the average per capita disposable income of Jinan), and highest (above average per capita disposable income of Jinan).

### AWAG Matrix Analysis

**Figure 1.**
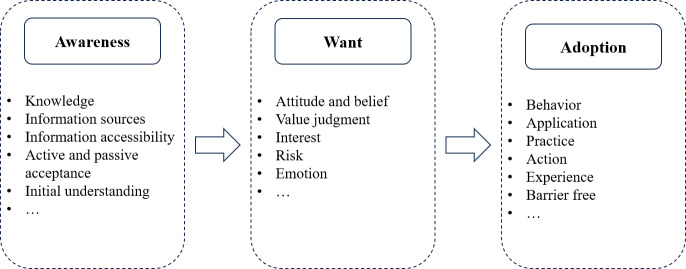
Schematic diagram of the association between awareness, want, and adoption.

This study used a matrix analysis based on the 3-stage consumer purchase decision model, which includes cognition, interest, and final decision [79]. As illustrated in Figure 1, individuals initially form an understanding or awareness of innovative things under the influence of both external factors and personal characteristics. On the basis of this awareness and their own needs, they develop a willingness to engage with the service. Ultimately, driven by their personal circumstances and external triggers, this willingness is transformed into actual adoption behavior [12,79].

To assess the digital divide in eHealth services, the study measured the percentage of participants who were aware of, wanted, and adopted eHealth services. In addition, the adoption gap rate of eHealth services was calculated to quantify the disparities in eHealth service adoption across the population [12], using the following formula:


$$Gap\;rate=\frac{min\left(Pr\left(A\left(X\right)\right),\mathrm{Pr}\left(W\left(x\right)\right)\right)-Pr\left(U\left(X\right)\right)}{min\left(Pr\left(A\left(X\right)\right),\mathrm{Pr}\left(W\left(x\right)\right)\right)}\times 100\%$$

where $Pr\left(A\left(X\right)\right)$ represents the awareness rate for eHealth service x;

$\mathrm{Pr}\left(W\left(x\right)\right)$ represents the want rate for eHealth service x; and

Pr(U(X) represents the adoption rate for eHealth service x.

Adoption gap rates range from 0% to 100%. Among those who were already aware of or wanted eHealth services, the adoption gap rate represents the percentage of individuals who have never used eHealth services. When the adoption rate is equal to the minimum value of the awareness rate and the want rate, the adoption gap rate is 0, indicating that all the people who have awareness or want for eHealth services have used them. Conversely, if no one has used eHealth services, the adoption gap rate is 100%.

On the basis of technology adoption lifecycle (bell curve) and the AWAG matrix method by Liang [80], this study grouped the innovators and the early majority stage and subdivided the 4 types of adopters into 3 adjusted adoption lifecycle accumulation rates of 15%, 50%, and 85%, including innovators or early adopters, early majority, late majority, and laggards [81]. The middle point of 50% divides both the awareness rates and the want rates into 2 levels. The AWAG matrix is thus divided into 4 primary categories: opened group, perception deficiency group, desire deficiency group, and closed group. According to Liang’s studies [80], the opened group includes individuals who are receptive to innovation, demonstrating a strong interest in seeking new information and exploring innovative ideas. Conversely, individuals classified as the closed group show little interest in innovation and are resistant to adopting new ideas. The perception deficiency group includes individuals who lack a strong awareness of innovation. While they remain open to new information and are willing to explore innovative things, they tend to lag behind in receiving new information. The desire deficiency group, on the other hand, consists of individuals who, despite being early recipients of new information, are not interested in innovation and even show resistance to trying something new. Using the cumulative rate of 15% or 85%, each category was further divided into 4 subcategories, that is, strong (S), generic (G), want-bias (Wb), and awareness-bias (Ab). The strong subgroup represents the most open, closed, perception deficient, or desire deficient group, whereas the generic subgroup represents the least in each respective category. The position of each circle on the matrix reflects the awareness and want rates for eHealth services, whereas the size of the circle indicates the adoption gap rate. Larger circles correspond to greater usage gaps for a specific eHealth service, implying lower overall utilization. For instance, an eHealth service in the Wb opened group has high awareness but lagging desire, suggesting a need for strategies that build trust and perceived usefulness rather than mere information campaigns. Conversely, an eHealth service in the Ab opened group has strong desire but low awareness, indicating that marketing and education efforts should be prioritized.

### Statistical Analysis

In the descriptive analysis, the mean and SD were used to summarize continuous variables with a normal distribution, whereas median and interquartile range (IQR) were adopted to describe those with a nonnormal distribution, including age, educational attainment, eHealth literacy, perceived usefulness, and perceived ease of use. For categorical variables, such as sex, marital status, place of residence, economic status, SRH, and chronic disease status, frequency and percentage were calculated to describe their distributions. Sample description and univariate analysis results are presented in Tables S1, S2, and S3 in the Multimedia Appendix 1.

To identify significant factors influencing the awareness of, want for, and adoption of eHealth services, binary logistic regression analysis was conducted, adjusting for potential confounding factors. The results were reported as odds ratios with corresponding 95% CIs, and statistical significance was set at a *P* value <.05 (2 sided). To account for potential clustering effects within hospitals, cluster-robust SEs were used in the logistic regression analysis. All statistical analyses were performed using Stata 17.0 (StataCorp LLC, USA), and the AWAG matrix figure was generated using MATLAB R2019b (The MathWorks, Inc, USA).

## Results

### Descriptive Statistics

Nearly half of the participants were male (611/1322, 46.2%). The median age was 53 years (IQR 40‐60). Of 1322 participants, 174 (13.2%) were unmarried. Rural residents accounted for 21.6% (285/1322), whereas the majority resided in urban areas (1037/1322, 78.4%). The median years of educational attainment was 9 (IQR 6‐13). The largest income group was those with the lowest income, representing 52.8% (698/1322) of the sample.

SRH showed a balanced distribution between negative (326/1322, 24.7%) and positive (388/1322, 29.3%) assessments, with the largest proportion reporting fair SRH (608/1322, 46.0%). Most inpatients had chronic diseases, accounting for 60.67% (802/1322) of the sample. Among the 3 digital technology factors, a median eHealth literacy score of 26 (IQR 16‐32) was reported, whereas the median scores of perceived usefulness and perceived ease of use were 16 (IQR 13‐17) and 14 (IQR 9‐16), respectively. The detailed information regarding inpatients is displayed in Table S1, S2, and S3 in Multimedia Appendix 1.

### AWAG Matrix Analysis Results

**Figure 2.**
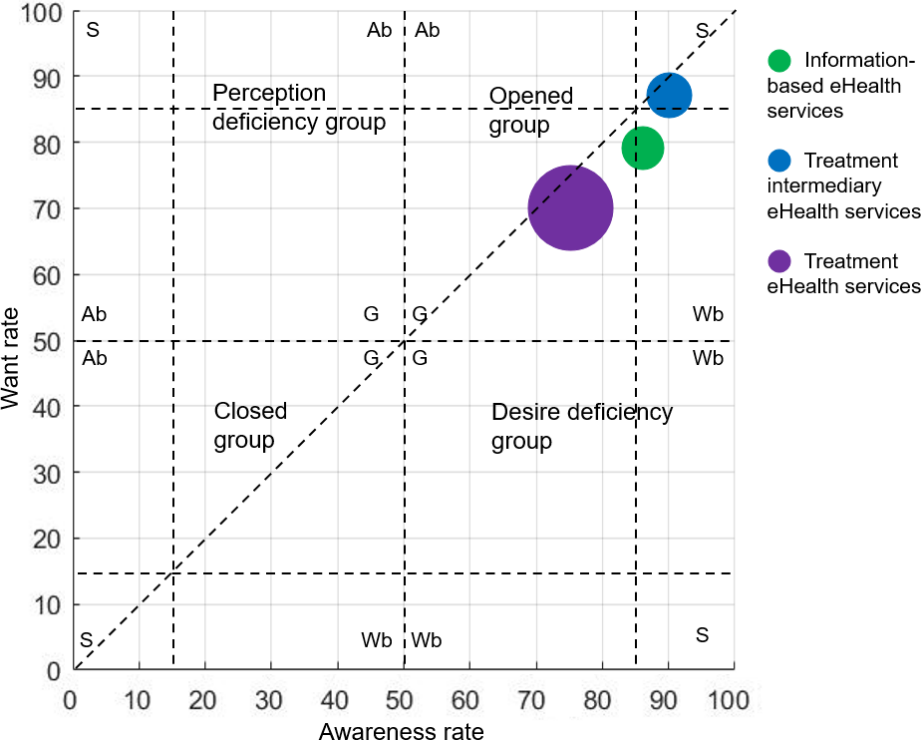
Awareness, want, and adoption gap matrix for information-based, treatment intermediary, and treatment eHealth services.

Table 3 presents the awareness, want, and adoption rates for eHealth services, including information-based, treatment intermediary, and treatment eHealth services. Overall, the awareness, want, and adoption rates for eHealth services were relatively high (all exceeding 50%), 1204 of 1322 inpatients (91.1%) had awareness of eHealth services, 88.4% (1169/1322) of them had a want for eHealth services, and 847 of 1322 inpatients (64.1%) adopted 1 or more of these services. The adoption gap ratio of eHealth services was 27.6%, categorizing it within the strong opened group. Among the 3 types of eHealth services, treatment intermediary eHealth services demonstrated the highest awareness (1182/1322, 89.4%), want (1142/1322, 86.4%), and adoption (753/1322, 57.0%) rates, with an adoption gap ratio of 34.1%. Conversely, treatment eHealth services showed the lowest rates, with awareness at 74.6% (986/1322), want at 69.9% (924/1322), and adoption at 23.5% (310/1322). The adoption gap ratio for treatment eHealth services was the highest, at 66.5%. As shown in Figure 2, the adoption gap ratio for treatment eHealth services (66.5%) was the highest and fell into the generic opened group. In contrast, information-based eHealth services had the lowest adoption gap ratio (32.1%), placing it within the Wb opened group.

**Table 3.** Awareness, want, and adoption gap of information-based, treatment intermediary, and treatment eHealth services.

Variables	Awareness, n (%)	Want, n (%)	Adoption, n (%)	Adoption gap ratio (%)	Region in awareness-want segment matrix^a^
eHealth services	1204 (91.1)	1169 (88.4)	847 (64.1)	27.6	O _ S
Information-based eHealth services	1128 (85.3)	1037 (78.4)	704 (53.3)	32.1	O _ Wb
Treatment intermediary eHealth services	1182 (89.4)	1142 (86.4)	753 (57.0)	34.1	O _ S
Treatment eHealth services	986 (74.6)	924 (69.9)	310 (23.1)	66.5	O _ G

a Groups are opened (O), desire deficiency (D), perception deficiency (P), and closed (C); regions are strong (S), generic (G), awareness-bias (Ab), and want-bias (Wb).

### Logistic Analysis of Awareness, Want, and Adoption on 3 eHealth Services

Table 4 presented logistic analysis results of 3 different eHealth services. Regarding general demographic characteristics, older patients were less likely to have awareness of, want for, and adoption of all 3 eHealth services (*P*<.001), except for want for and adoption of treatment eHealth services (*P*>.05). Inpatients living in rural areas were less likely to have a want for all 3 eHealth services (*P*<.05). Educational attainment displayed a significant positive association with awareness and adoption of these 3 services (*P*<.01). Compared to inpatients with the lowest income, inpatients with middle income were more likely to have awareness of information-based and treatment intermediary eHealth services, whereas those with the highest were more likely to have a want for treatment intermediary and treatment eHealth services (*P*<.05).

**Table 4.** Logistic regression results for awareness of, want for, and adoption of information-based, treatment intermediary, and treatment eHealth services.

Variables	Information-based eHealth service, OR^a^ (95% CI)			Treatment intermediary eHealth services, OR (95% CI)			Treatment eHealth services, OR (95% CI)		
	Model 1	Model 2	Model 3	Model 4	Model 5	Model 6	Model 7	Model 8	Model 9
	Awareness^b^	Want^c^	Adoption^d^	Awareness	Want	Adoption	Awareness	Want	Adoption
Gender									
Female (ref: male)	1.392 (0.720-2.689)	1.140 (0.857- 1.517)	0.895 (0.677- 1.183)	1.142 (0.631-2.068)	0.896 (0.537- 1.495)	1.003 (0.755- 1.331)	1.310^e^ (1.097- 1.564)	0.863 (0.651- 1.142)	1.084 (0.937- 1.253)
Age	0.927^f^ (0.904-0.951)	0.956^f^ (0.947- 0.965)	0.956^f^ (0.934- 0.978)	0.940^f^ (0.911-0.970)	0.940^f^ (0.926- 0.954)	0.950^f^ (0.932- 0.969)	0.943^f^ (0.922- 0.964)	0.989 (0.961- 1.018)	0.994 (0.987- 1.001)
Marital status									
Single (ref: married)	0.598 (0.317-1.129)	0.896 (0.588- 1.367)	0.675^f^ (0.544- 0.838)	0.573 (0.267-1.228)	0.784 (0.548- 1.120)	1.078 (0.739- 1.573)	0.671^f^ (0.603- 0.747)	1.101 (0.785- 1.544)	1.502 (0.525- 4.297)
Place of residence									
Rural (ref: urban)	0.447^f^ (0.299-0.669)	0.524^e^ (0.363- 0.756)	0.559 (0.308- 1.013)	0.604 (0.312-1.171)	0.350^g^ (0.155- 0.793)	0.428^e^ (0.237- 0.772)	0.605 (0.227- 1.613)	0.583^f^ (0.554- 0.613)	0.797 (0.440- 1.444)
Educational attainment	1.104^f^ (1.066-1.142)	1.022 (0.969- 1.077)	1.034^f^ (1.018- 1.050)	1.118^f^ (1.105-1.131)	1.068 (0.992- 1.149)	1.106^f^ (1.073- 1.140)	1.089^e^ (1.028- 1.153)	0.993 (0.979- 1.007)	1.031^g^ (1.001- 1.063)
Income									
Middle (ref: lowest)	1.424^f^ (1.277-1.588)	1.599 (0.568- 4.507)	1.304 (0.679- 2.504)	1.205^g^ (1.029-1.412)	0.901 (0.514- 1.578)	1.057 (0.810- 1.380)	0.726 (0.296- 1.785)	1.670 (0.884- 3.153)	0.919 (0.592- 1.427)
Highest (ref: lowest)	1.426 (0.805-2.527)	2.237^f^ (1.758- 2.847)	1.231 (0.841- 1.800)	2.309 (0.938-5.683)	2.206 (0.998- 4.877)	1.471 (0.919- 2.355)	0.880 (0.266- 2.912)	1.399^f^ (1.185- 1.651)	0.954 (0.733- 1.243)
SRH^h^									
Fair (ref: negative)	0.595^f^ (0.475-0.746)	0.355^e^ (0.189- 0.667)	0.597^g^ (0.373- 0.953)	0.480^f^ (0.417-0.552)	0.471^f^ (0.386- 0.576)	0.581^e^ (0.418- 0.806)	0.796 (0.543- 1.167)	0.630^f^ (0.502- 0.791)	0.689^f^ (0.594- 0.799)
Positive (ref: negative)	0.360^f^ (0.328-0.396)	0.287^f^ (0.248- 0.332)	0.319^e^ (0.152- 0.671)	0.450 (0.177-1.143)	0.491^e^ (0.318- 0.757)	0.485^f^ (0.421- 0.558)	0.640 (0.343- 1.195)	0.523^g^ (0.273- 1.001)	0.510^f^ (0.430- 0.604)
Chronic disease									
Yes (ref：no)	0.724 (0.427-1.228)	1.265 (0.529- 3.024)	1.275 (0.600- 2.712)	0.693^g^ (0.509-0.944)	1.003 (0.488- 2.061)	0.789 (0.473- 1.315)	0.550^g^ (0.328- 0.921)	0.829 (0.472- 1.457)	0.832^e^ (0.742- 0.934)
eHealth literacy	1.133^f^ (1.097-1.171)	1.050^f^ (1.041- 1.058)	1.048^e^ (1.016- 1.082)	1.082^f^ (1.059-1.106)	0.963 (0.916- 1.013)	0.992 (0.960- 1.025)	1.114^f^ (1.091- 1.138)	1.018 (0.979- 1.060)	1.062^g^ (1.006- 1.120)
Perceived usefulness										—^i^	1.365^f^ (1.220- 1.528)	1.202^f^ (1.180- 1.225)	—	1.379^f^ (1.287- 1.479)	1.154^f^ (1.109- 1.202)	—	1.335^f^ (1.258- 1.417)	1.107^f^ (1.088- 1.126)
Perceived ease of use	—	1.023 (0.854- 1.226)	1.046 (0.966- 1.132)	—	1.108 (0.938- 1.310)	1.110^g^ (1.021- 1.207)	—	1.069 (0.929- 1.231)	1.095^g^ (1.005- 1.193)									

a OR: odds ratio.

b Model 1 (awareness), Model 4 (awareness), Model 7 (awareness): logistic regression model adjusted for sex, age, marital status, place of residence, educational attainment, income, SRH, chronic disease, and eHealth literacy.

c Model 2 (want) adds perceived usefulness and ease of use to the variables in model 1; model 5 (want) adds perceived usefulness and ease of use to the variables in model 2; model 8 (want) adds perceived usefulness and ease of use to the variables in model 7.

d Model 3 (adoption) includes all variables from model 2; model 6 (adoption) includes all variables from model 5; model 9 (adoption) includes all variables from model 8.

e *P*<.01.

f P<.001.

g *P*<.05

h SRH: self-rated health.

i Not applicable.

Health status was an important factor that influenced awareness, want, and adoption in eHealth services among inpatients. Inpatients with more positive SRH were less likely to have awareness of, want for, and adoption of 3 services (*P*<.05). Having chronic diseases was only significantly negative with awareness of treatment intermediary and treatment eHealth services and adoption of treatment eHealth services (*P*<.01).

Among digital technology factors, eHealth literacy demonstrated a positive correlation with awareness of all 3 services (*P*<.001), and it had a favorable influence on want for information-based eHealth services and adoption of information-based and treatment eHealth services (*P*<.05). Perceived usefulness exerted a positive effect on both want for and adoption of 3 services (*P*<.001). Finally, perceived ease of use had a positive influence on the adoption of treatment intermediary and treatment eHealth services (*P*<.05).

Among inpatients, age, living in rural areas, and better SRH negatively influenced awareness, want, and adoption in eHealth services, but educational attainment, eHealth literacy, perceived usefulness, and perceived ease of use were positively associated with these outcomes. In addition, the influence of these factors differed depending on the specific type of eHealth service. The logistic analysis results for awareness of, want for, and adoption of eHealth services were presented in Table S4 in Multimedia Appendix 1.

## Discussion

### Principal Findings

The findings of this study confirmed the existence of a digital divide in eHealth services among information-based, treatment intermediary, and treatment eHealth services. In addition, the study further demonstrated a reciprocal relationship between the want and awareness rate, suggesting that a higher awareness rate may stimulate greater want rate for eHealth services, and consequently, adoption rate of eHealth services also tends to increase with elevated awareness and want rates. These observations align with the findings of Te-Hsin Liang’s research on 2 types of eHealth services in Taiwan [80]. However, it is important to note that these findings are based on a sample from 3 hospitals in a single city in China, which may limit the generalizability of the results to other regions or populations.

In the AWAG matrix, information-based, treatment intermediary, and treatment eHealth services were all categorized within the opened group. Compared to other studies, inpatients in this research exhibited relatively high levels of awareness and want for these services. The internet, recognized as a rapidly expanding platform for health information dissemination [82,83], has emerged as a pivotal platform for accessing comprehensive health-related data, effectively catering to diverse health care stakeholders [83]. Inpatients, specifically, rely heavily on the internet to remain informed about their health status and to educate themselves on disease treatments [84]. While treatment intermediary eHealth services were positioned within the strong subgroup, information-based eHealth services were categorized in the Wb subgroup. Previous research highlights an increasing reliance on internet searches when addressing health concerns [85]. However, challenges persist regarding the quality of online health information [82]. Overuse or inappropriate use of information-based eHealth services can lead to exaggerated or misinterpreted adverse symptoms, potentially heightening health anxiety or fear [86,87]. Governmental support and hospital-led initiatives to raise awareness and promote the use of these services are instrumental in their effectiveness [88,89]. The adoption gap between treatment intermediary and information-based eHealth services was comparable, indicating similar potential for increased utilization of treatment intermediary eHealth services. This highlights the importance of developing a robust maintenance strategy. Several pivotal factors contributing to the digital divide in the adoption of such services have been identified through research, including inadequate staffing levels in hospitals, outdated medical technology, system implementation challenges, and the overarching health care environment [90]. However, thanks to policy support and advancements in medical technology [91], the adoption gap rate for eHealth services in this study is notably lower compared to that reported in comparable research endeavors [80].

Furthermore, treatment eHealth services fell within the generic subgroup characterized by relatively lower rates of awareness and want compared to the other 2 service categories. The late introduction of treatment eHealth services has led to limited awareness among inpatients [92]. Some studies have also found that this lack of awareness is further exacerbated by the higher eHealth literacy requirements for using treatment eHealth services [93], which contributes to lower want rates [60]. Similar findings were observed in this study, showing that eHealth literacy positively influenced awareness of treatment eHealth services, while the want for these services remained relatively low. Notably, the adoption gap rate for treatment eHealth services was 66.5%, suggesting ample prospects for augmenting their adoption. Addressing barriers such as inadequate awareness and low eHealth literacy could play a pivotal role in narrowing this gap and enhancing the utilization of treatment eHealth services.

Factors influencing all 3 eHealth services consistently indicated that younger participants were more likely to be aware of and want eHealth services, whereas older participants demonstrated a greater propensity to adopt them. The decline in physical function commonly observed in older patients presents challenges in using electronic devices [16], thereby perpetuating the digital divide. Furthermore, apart from the lack of access to digital devices [94], the process of learning to use the internet may evoke feelings of anxiety or embarrassment among older adults [95]. Cao et al [96] demonstrated that weekly online and offline knowledge and psychological interventions significantly improved the knowledge, willingness, confidence, and usage of internet medical services among older patients with chronic diseases in China. This suggests that the digital divide in eHealth services, driven by nonmaterial barriers among the older, can be mitigated through targeted knowledge training and mindset improvement, particularly in middle- and high-income countries [97].

Rural participants were less likely to be aware of, want, and adopt eHealth services, consistent with previous findings and further substantiating the digital divide [6,98]. Likewise, negative SRH has a negative effect on eHealth services’ awareness, want, and adoption. This finding aligns with Andersen and Newman’s individual determinants of disease levels [99], which suggest that it is the direct cause of want for and adoption of eHealth services among inpatients in this study. A study on telemedicine in the United States also found that residing in rural areas and access to broadband had a greater impact on the use of telemedicine than other socioeconomic factors, which highlights the importance of understanding not only broadband access but also the broader relationship between the rural environment and telemedicine use [100].

The impact of income and educational attainment on eHealth services varied notably depending on the specific type of service. Compared to treatment intermediary eHealth services [42,101], information-based eHealth services typically have lower costs and are easier to use [102]. Similarly, this study also identified a significant positive effect of educational attainment on awareness and adoption of all 3 types of eHealth services. The research by Limbu and Huhmann [103] and Reinecke et al [104] further support these findings, highlighting that both income and education serve as important enablers for access to more expensive or complex eHealth services, regardless of whether in high-income or middle- and low-income countries, in which reforms are expected to address the digital divide. However, inpatients with poor SRH and chronic diseases were likely to want or adopt all 3 eHealth services, especially treatment services. Some studies have indicated that individuals with poor health conditions or chronic diseases exhibit a heightened demand for eHealth services that can address health issues and provide monitoring, potentially overcoming barriers to usage [2,72].

Moreover, higher eHealth literacy implies a stronger ability to acquire information and benefit from eHealth services [105]. Beyond its positive influence on awareness, eHealth literacy was also significantly associated with the want for information-based eHealth services and the adoption of treatment eHealth services. Specifically, younger patients with higher eHealth literacy scores demonstrated a greater likelihood of desiring and adopting these services, aligning with the findings of other studies conducted globally [106-108].

Perceived usefulness has a significant positive impact on eHealth services’ demand and use, consistent with previous studies [109,110]. However, in this study, perceived ease of use only has a positive impact on the adoption of eHealth services. This result aligns with prior research, suggesting that perceived ease of use indirectly influences the intention to use eHealth services through perceived usefulness, rather than exerting a direct effect [109]. In addition, a survey by Wu et al [111] on inpatient participation in telemedicine in Toronto reveals that perceived usefulness, along with prior positive experiences, was a key factor driving participants’ willingness to engage in various telemedicine services. These results further underscore the importance of enhancing patients’ experiences and perceptions, as doing so can foster greater use of eHealth and help address the digital divide.

This study holds significant implications for eHealth policies and practices. The AWAG matrix analysis shows a digital divide in eHealth exists among inpatients in Jinan, considering various adoption stages and service types. Future digital health initiatives should avoid adopting a one-size-fits-all strategy and, instead, aim to achieve digital health equity through tailored services that account for varying user characteristics and needs. Information-based services were characterized by want bias. To enhance the credibility of information, it is recommended to introduce doctor certification and user evaluation systems, whereas targeted promotional efforts should be directed toward rural and low-education groups, using community health lectures, bulletin boards, and broadcasts in primary health care institutions. These low-cost measures can be followed by low- and middle-income countries. For treatment intermediary services with higher awareness, want, and adoption, it is essential to optimize operational processes, improve system stability, and enhance response speed to elevate the user experience. This can be achieved by simplifying appointment scheduling, payment procedures, and query results. However, the awareness, want, and adoption rate of medical treatment remain relatively low. It is crucial for the government and hospitals to collaborate to promote policy support. Therefore, collaboration between the government and health care institutions is critical to foster policy support. For example, governments should include eHealth or telemedicine services within the scope of medical insurance reimbursement and encourage both doctors and patients to use online follow-up services.

### Limitations

This study acknowledges several limitations. First, it uses a cross-sectional survey design, which inherently restricts the ability to analyze influence pathways and establish causality among the relevant variables. Future studies adopting a longitudinal design would be more appropriate to address this limitation. Second, the sample population consists of inpatients from 3 hospitals in Jinan, Shandong Province, who inherently exhibit a certain level of medical service demand. Consequently, caution should be exercised when generalizing the findings to other populations. Finally, as a retrospective study, it is susceptible to recall bias, which could impact the accuracy of the data and results.

### Conclusions

This study delves into inpatients’ awareness of, want for, and adoption of eHealth services by using a matrix analysis conducted among inpatients at 3 hospitals in Jinan, China. Information-based eHealth services, categorized within the opened Wb group in the AWAG matrix, revealed a significant digital divide in their usage. To address this, targeted strategies should focus on enhancing privacy protection and improving the perceived ease of use of these services, which could help sustain and gradually increase demand. Treatment intermediary eHealth services, classified within the opened strong group, also demonstrated a substantial digital divide in adoption, necessitating further attention. Finally, treatment eHealth services, positioned in the opened generic group, continue to face significant adoption challenges, with both awareness and want requiring improvement. To bridge these gaps, initiatives such as frequent public awareness campaigns and improving response efficiency need to be implemented to enhance awareness and foster sustained demand. The findings also underscore the diverse health care needs of individuals, shaped by factors such as educational attainment and place of residence. These differences necessitate comprehensive strategies, particularly in addressing the challenges older adults face in navigating internet technologies.

## Supplementary material

10.2196/72297 Multimedia Appendix 1 Basic descriptive statistics, sensitivity analysis, and survey questionnaire.

## Abbreviations

Ab: awareness-bias
AWAG: awareness, want, and adoption gap
eHEALS: the eHealth literacy scale
IQR: interquartile range
SRH: self-rated health
Wb: want-bias
