# Supplementary material for: Digital Divide in Awareness, Want, and Adoption Across Diverse eHealth Services: Cross-Sectional Survey of Inpatients in Jinan, China
Source: J Med Internet Res. 2025 Oct 30;27:e72297. doi: 10.2196/72297 (PMC12574752; doi:10.2196/72297)
Supplement: Multimedia Appendix 1 [file jmir-v27-e72297-s001.docx]

**Supplemental material**

**Table S1 Description and univariate analysis results for awareness of, want for, and adoption of information-based eHealth services^a,b^**

| **Variables** | **All** | **Awareness** |  |  | **Want** |  |  | **Adoption** |  |  |
| --- | --- | --- | --- | --- | --- | --- | --- | --- | --- | --- |
|  |  | **Yes** | **No** | **Z/χ^2^** | **Yes** | **No** | **Z/χ^2^** | **Yes** | **No** | **Z/χ^2^** |
| **Gender, n (%)** |  |  |  | 3.124 |  |  | 2.713 |  |  | 1.028 |
| Male | 611 (46.22) | 510 (83.47) | 101 (16.53) |  | 467 (76.43) | 144 (23.57) |  | 371 (45.13%) | 240 (48.00%) |  |
| Female | 711 (53.78) | 618 (86.92) | 93 (13.08) |  | 570 (80.17) | 141 (19.83) |  | 451 (54.87%) | 260 (52.00%) |  |
| **Age (y), median (IQR)** | 53 (40-64) | 50 (37-60) | 67 (59-74) | 14.672^***^ | 49 (37-59) | 65 (57-71) | 14.922^***^ | 46 (35-56) | 62 (54-70) | 17.596*** |
| **Marital status, n (%)** |  |  |  | 0.635 |  |  | 0.797 |  |  | 3.288 |
| Married | 1148 (86.84) | 983 (85.63) | 165 (14.37) |  | 896 (78.05) | 252 (21.95) |  | 703 (85.52%) | 445 (89.00%) |  |
| Unmarried | 174 (13.16) | 145 (83.33) | 29 (16.67) |  | 141 (81.03) | 33 (18.97) |  | 119 (14.48%) | 55 (11.00%) |  |
| **Place of residence, n (%)** |  |  |  | 49.375^***^ |  |  | 28.041^***^ |  |  | 42.391*** |
| Urban | 1037 (78.44) | 922 (88.91) | 115 (11.09) |  | 846 (81.58) | 191 (18.42) |  | 692 (84.18%) | 345 (69.00%) |  |
| Rural | 285 (21.56) | 206 (72.28) | 79 (27.72) |  | 191 (45.98) | 94 (54.02) |  | 130 (15.82%) | 155 (31.00%) |  |
| **Educational attainment (y), median (IQR)** | 9 (6-13) | 10 (8-15) | 6 (3-9) | -12.587^***^ | 11 (8-15) | 8 (5-9) | -10.703^***^ | 12 (9-15) | 8.5 (5-9) | -13.479*** |
| **Income, n (%)** |  |  |  | 22.541^***^ |  |  | 6.443^*^ |  |  | 22.831*** |
| Lowest | 698 (52.80) | 612 (87.68) | 86 (12.32) |  | 558 (79.94) | 140 (20.06) |  | 464 (56.45%) | 234 (46.80%) |  |
| Middle | 498 (37.67) | 426 (85.54) | 72 (14.46) |  | 391 (78.51) | 107 (21.49) |  | 302 (36.74%) | 196 (39.20%) |  |
| Highest | 126 (9.53) | 90 (71.43) | 36 (28.57) |  | 88 (69.84) | 38 (30.16) |  | 56 (6.81%) | 70 (14.00%) |  |
| **SRH, n (%)** |  |  |  | 5.104 |  |  | 3.830 |  |  | 8.198* |
| Negative | 326 (24.66) | 267 (81.90) | 59 (18.10) |  | 255 (78.22) | 71 (21.78) |  | 183 (22.26%) | 143 (28.60%) |  |
| Fair | 608 (45.99) | 520 (85.53) | 88 (14.47) |  | 465 (76.48) | 143 (23.52) |  | 381 (46.35%) | 227 (45.40%) |  |
| Positive | 388 (29.35) | 341 (87.89) | 47 (12.11) |  | 317 (81.70) | 71 (18.30) |  | 258 (31.39%) | 130 (26.00%) |  |
| **Chronic disease, n (%)** |  |  |  | 22.319^***^ |  |  | 44.819^***^ |  |  | 59.302*** |
| No | 520 (39.33) | 714 (89.03) | 88 (10.97) |  | 678 (84.54) | 124 (15.46) |  | 565 (68.73%) | 237 (47.40%) |  |
| Yes | 802 (60.67) | 414 (79.62) | 106 (20.38) |  | 359 (69.04) | 161 (30.96) |  | 257 (31.27%) | 263 (52.60%) |  |
| **eHealth literacy score, median (IQR)** | 26 (16-32) | 28 (21-33) | 15 (8-21) | -15.731^***^ | 29 (22-33) | 16 (8-24) | -16.659^***^ | 30 (24-34) | 16 (10-24) | -18.087*** |
| **Perceived usefulness score, median (IQR)** | 16 (13-17) |  |  |  | 16 (15-18) | 12 (8-14) | -17.887^***^ | 16 (15-18) | 13 (11-16) | -16.738*** |
| **Perceived ease of use score, median (IQR)** | 14 (9-16) |  |  |  | 16 (12-17) | 8 (4-12) | -16.617^***^ | 16 (12-18) | 9 (6-13) | -18.397*** |

^a^Regarding the statistical description of variables, this study used median and Interquartile Range (IQR) for continuous variables and frequency (n) and percentage for categorical variables; *P* values were calculated using rank-sum tests for continuous variables and χ^2^ tests for categorical variables.

^b^**p* < 0.05, ***p* < 0.01, *p* < 0.001.

**Table S2 Description and univariate analysis results for awareness of, want for, and adoption of treatment intermediary eHealth services^a,b^**

| **Variables** | **All** | **Awareness** |  |  | **Want** |  |  | **Adoption** |  |  |
| --- | --- | --- | --- | --- | --- | --- | --- | --- | --- | --- |
|  |  | **Yes** | **No** | **Z/χ^2^** | **Yes** | **No** | **Z/χ^2^** | **Yes** | **No** | **Z/χ^2^** |
| **Gender, n (%)** |  |  |  | 0.349 |  |  | 0.001 |  |  | 0.337 |
| Male | 611 (46.22) | 543 (88.87) | 68 (11.13) |  | 528 (86.42) | 83 (13.58) |  | 460 (45.77%) | 151 (47.63%) |  |
| Female | 711 (53.78) | 639 (89.87) | 72 (10.13) |  | 614 (86.36) | 97 (13.64) |  | 545 (54.23%) | 166 (52.37%) |  |
| **Age (y), median (IQR)** | 53 (40-64) | 51 (38-61) | 66.5 (59-73) | 11.824^***^ | 51 (37-60) | 66 (59-72) | 13.490^***^ | 48 (36-59) | 64 (57-71) | 15.816*** |
| **Marital status, n (%)** |  |  |  | 0.893 |  |  | 0.096 |  |  | 2.763 |
| Married | 1148 (86.84) | 1030 (89.72) | 118 (10.28) |  | 993 (86.50) | 155 (13.50) |  | 864 (85.97%) | 284 (89.59%) |  |
| Unmarried | 174 (13.16) | 152 (87.36) | 22 (12.64) |  | 149 (85.63) | 25 (14.37) |  | 141 (14.03%) | 33 (10.41%) |  |
| **Place of residence, n (%)** |  |  |  | 24.598^***^ |  |  | 34.674^***^ |  |  | 65.488*** |
| Urban | 1037 (78.44) | 950 (91.61) | 87 (8.39) |  | 926 (89.30) | 111 (10.70) |  | 840 (83.58%) | 197 (62.15%) |  |
| Rural | 285 (21.56) | 232 (81.40) | 53 (18.60) |  | 216 (75.79) | 69 (24.21) |  | 165 (16.42%) | 120 (37.85%) |  |
| **Educational attainment (y), median (IQR)** | 9 (6-13) | 9 (7-14) | 6 (3.5-9) | -10.431^***^ | 10 (7-15) | 6 (5-9) | -10.302^***^ | 12 (9-15) | 6 (5-9) | -14.108*** |
| **Income, n (%)** |  |  |  | 3.406 |  |  | 5.851 |  |  | 15.200** |
| Lowest | 698 (52.80) | 633 (90.69) | 65 (9.31) |  | 617 (88.40) | 81 (11.60) |  | 555 (55.22%) | 143 (45.11%) |  |
| Middle | 498 (37.67) | 441 (88.55) | 57 (11.45) |  | 422 (84.74) | 76 (15.26) |  | 369 (36.72%) | 129 (40.69%) |  |
| Highest | 126 (9.53) | 108 (85.71) | 18 (14.29) |  | 103 (81.75) | 23 (18.25) |  | 81 (8.06%) | 45 (14.20%) |  |
| **SRH, n (%)** |  |  |  | 3.976 |  |  | 5.135 |  |  | 7.515* |
| Negative | 326 (24.66) | 289 (88.65) | 37 (11.35) |  | 278 (85.28) | 48 (14.72) |  | 238 (23.68%) | 88 (27.76%) |  |
| Fair | 608 (45.99) | 536 (88.16) | 72 (11.84) |  | 516 (84.87) | 92 (15.13) |  | 453 (45.07%) | 155 (48.90%) |  |
| Positive | 388 (29.35) | 357 (92.01) | 31 (7.99) |  | 348 (89.69) | 40 (10.31) |  | 314 (31.24%) | 74 (23.34%) |  |
| **Chronic disease, n (%)** |  |  |  | 8.498^**^ |  |  | 26.232^***^ |  |  | 21.682*** |
| No | 520 (39.33) | 733 (91.40) | 69 (8.60) |  | 724 (90.27) | 78 (9.73) |  | 645 (64.18%) | 157 (49.53%) |  |
| Yes | 802 (60.67) | 449 (86.35) | 71 (13.65) |  | 418 (80.38) | 102 (19.62) |  | 360 (35.82%) | 160 (50.47%) |  |
| **eHealth literacy score, median (IQR)** | 26 (16-32) | 28 (19-32) | 16 (8-24) | -11.520^***^ | 28 (20-32) | 16 (8-24) | -13.109^***^ | 29 (22-33) | 16 (8-24) | -14.742*** |
| **Perceived usefulness score, median (IQR)** | 16 (13-17) |  |  |  | 16 (14-18) | 12 (8-13) | -14.990^***^ | 16 (14-18) | 12 (9-16) | -13.642*** |
| **Perceived ease of use score, median (IQR)** | 14 (9-16) |  |  |  | 15 (11-17) | 8 (4-12) | -14.125^***^ | 16 (12-17) | 8 (4-12) | -15.959*** |

^a^Regarding the statistical description of variables, this study used median and Interquartile Range (IQR) for continuous variables and frequency (n) and percentage for categorical variables; *P* values were calculated using rank-sum tests for continuous variables and χ^2^ tests for categorical variables.

^b^**p* < 0.05, ***p* < 0.01, *p* < 0.001.

**Table S3 Description and univariate analysis results for awareness of, want for, and adoption of treatment eHealth services^a,b^**

| **Variables** | **All** | **Awareness** |  |  | **Want** |  |  | **Adoption** |  |  |
| --- | --- | --- | --- | --- | --- | --- | --- | --- | --- | --- |
|  |  | **Yes** | **No** | **Z/χ^2^** | **Yes** | **No** | **Z/χ^2^** | **Yes** | **No** | **Z/χ^2^** |
| **Gender, n (%)** |  |  |  | 5.034^*^ |  |  | 0.055 |  |  | 1.791 |
| Male | 611 (46.22) | 438 (71.69) | 173 (28.31) |  | 429 (70.21) | 182 (29.79) |  | 176 (43.46%) | 435 (47.44%) |  |
| Female | 711 (53.78) | 548 (77.07) | 163 (22.93) |  | 495 (69.62) | 216 (30.38) |  | 229 (56.54%) | 482 (52.56%) |  |
| **Age (y), median (IQR)** | 53 (40-64) | 48 (36-59) | 65 (57-71) | 16.922^***^ | 49 (37-60) | 60 (51-69) | 10.931^***^ | 44 (33-54) | 57 (45-66) | 11.773*** |
| **Marital status, n (%)** |  |  |  | 0.893 |  |  | 0.096 |  |  |  |
| Married | 1148 (86.84) | 1030 (89.72) | 118 (10.28) |  | 993 (86.50) | 155 (13.50) |  | 328 (80.99%) | 820 (89.42%) |  |
| Unmarried | 174 (13.16) | 152 (87.35) | 22 (12.64) |  | 149 (85.63) | 25 (14.37) |  | 77 (19.01%) | 97 (10.58%) |  |
| **Place of residence, n (%)** |  |  |  | 46.863^***^ |  |  | 26.336^***^ |  |  | 19.339*** |
| Urban | 1037 (78.44) | 818 (78.89) | 219 (21.12) |  | 760 (73.29) | 277 (26.71) |  | 348 (85.93%) | 689 (75.14%) |  |
| Rural | 285 (21.56) | 168 (58.95) | 117 (41.05) |  | 164 (57.54) | 121 (42.46) |  | 57 (14.07%) | 228 (24.86%) |  |
| **Educational attainment (y), median (IQR)** | 9 (6-13) | 12 (9-15) | 6 (5-9) | -14.788^***^ | 10 (8-15) | 9 (6-12) | -8.336^***^ | 12 (9-16) | 9 (6-12) | -10.315*** |
| **Income, n (%)** |  |  |  | 47.946^***^ |  |  | 9.579^**^ |  |  | 27.419*** |
| Lowest | 698 (52.80) | 570 (81.66) | 128 (18.34) |  | 494 (70.77) | 204 (29.23) |  | 255 (62.96%) | 443 (48.31%) |  |
| Middle | 498 (37.67) | 345 (69.28) | 153 (30.72) |  | 357 (71.69) | 141 (28.31) |  | 128 (31.60%) | 370 (40.35%) |  |
| Highest | 126 (9.53) | 71 (56.35) | 55 (43.65) |  | 73 (57.94) | 53 (42.06) |  | 22 (5.43%) | 104 (11.34%) |  |
| **SRH, n (%)** |  |  |  | 23.187^***^ |  |  | 2.026 |  |  | 10.110** |
| Negative | 326 (24.66) | 215 (65.95) | 111 (34.05) |  | 224 (68.71) | 102 (31.29) |  | 82 (20.25%) | 244 (26.61%) |  |
| Fair | 608 (45.99) | 454 (74.67) | 154 (25.33) |  | 418 (68.75) | 190 (31.25) |  | 183 (45.19%) | 425 (46.35%) |  |
| Positive | 388 (29.35) | 317 (81.70) | 71 (18.30) |  | 282 (72.68) | 106 (27.32) |  | 140 (34.57%) | 248 (27.05%) |  |
| **Chronic disease, n (%)** |  |  |  | 20.294^***^ |  |  | 26.232^***^ |  |  | 15.567*** |
| No | 520 (39.33) | 633 (78.93) | 169 (21.07) |  | 724 (90.27) | 78 (9.73) |  | 278 (68.64%) | 524 (57.14%) |  |
| Yes | 802 (60.67) | 353 (67.88) | 167 (32.12) |  | 418 (80.38) | 102 (19.62) |  | 127 (31.36%) | 393 (42.86%) |  |
| **eHealth literacy score, median (IQR)** | 26 (16-32) | 29 (24-34) | 16 (8-24) | -18.344^***^ | 29 (23-34) | 17 (10-26) | -14.681^***^ | 32 (28-36) | 24 (16-30) | -15.540*** |
| **Perceived usefulness score, median (IQR)** | 16 (13-17) |  |  |  | 16 (15-18) | 12.5 (10-16) | -16.591^***^ | 17 (16-19) | 15 (12-16) | -13.175*** |
| **Perceived ease of use score, median (IQR)** | 14 (9-16) |  |  |  | 16 (12-17) | 9 (6-14) | -14.944^***^ | 16 (15-19) | 12 (8-16) | -15.411*** |

^a^Regarding the statistical description of variables, this study used median and Interquartile Range (IQR) for continuous variables and frequency (n) and percentage for categorical variables; *P* values were calculated using rank-sum tests for continuous variables and χ^2^ tests for categorical variables.

^b^**p* < 0.05, ***p* < 0.01, *p* < 0.001.

**Table S4** **Logistic regression results for awareness of, want for and adoption of eHealth services**

| **Variables** |  | **Model 1^a^** |  |  | **Model 2^b^** |  |  | **Model 3^c^** |  |
| --- | --- | --- | --- | --- | --- | --- | --- | --- | --- |
|  |  | **Awareness** |  |  | **Want** |  |  | **Adoption** |  |
|  |  | **Odds ratio^d^** | **95% CI** |  | **Odds ratio** | **95% CI** |  | **Odds ratio** | **95% CI** |
| **Sex** |  |  |  |  |  |  |  |  |  |
| Female (ref: male) |  | 1.200 | [0.668, 2.155] |  | 1.287 | [0.696, 2.380] |  | 0.970 | [0.758, 1.241] |
| **Age** |  | 0.932*** | [0.904, 0.962] |  | 0.930*** | [0.923, 0.938] |  | 0.945*** | [0.917, 0.974] |
| **Marital status** |  |  |  |  |  |  |  |  |  |
| Single (ref: married) |  | 0.572 | [0.323, 1.015] |  | 0.763 | [0.499, 1.167] |  | 1.014 | [0.783, 1.313] |
| **Place of residence** |  |  |  |  |  |  |  |  |  |
| Rural (ref: urban) |  | 0.510* | [0.289, 0.898] |  | 0.378** | [0.181, 0.790] |  | 0.387*** | [0.259, 0.579] |
| **Educational attainment** |  | 1.116*** | [1.089, 1.144] |  | 1.085** | [1.023, 1.151] |  | 1.087*** | [1.046, 1.131] |
| **Income** |  |  |  |  |  |  |  |  |  |
| Middle (ref: lowest) |  | 1.453 | [0.888, 2.378] |  | 1.341 | [0.723, 2.487] |  | 1.104 | [0.721, 1.690] |
| Highest (ref: lowest) |  | 2.296 | [0.810, 6.511] |  | 2.586 | [0.997, 6.704] |  | 1.312* | [1.026, 1.677] |
| **SRH^e^** |  |  |  |  |  |  |  |  |  |
| Fair (ref: negative) |  | 0.583** | [0.391, 0.868] |  | 0.543*** | [0.413, 0.713] |  | 0.582*** | [0.495, 0.683] |
| Positive (ref: negative) |  | 0.540 | [0.245, 1.191] |  | 0.626 | [0.326, 1.200] |  | 0.386*** | [0.280, 0.533] |
| **Chronic disease** |  |  |  |  |  |  |  |  |  |
| Yes (ref：no) |  | 0.688** | [0.549, 0.862] |  | 0.802 | [0.406, 1.584] |  | 0.650 | [0.348, 1.213] |
| **eHealth literacy score** |  | 1.086*** | [1.055, 1.118] |  | 0.980 | [0.959, 1.001] |  | 0.998 | [0.980, 1.016] |
| **Perceived usefulness** |  |  |  |  | 1.457*** | [1.373, 1.547] |  | 1.193*** | [1.134, 1.256] |
| **Perceived ease of use** |  |  |  |  | 1.074 | [0.955, 1.207] |  | 1.110*** | [1.059, 1.164] |

^a^Model 1 (Awareness): logistic regression model adjusted for sex, age, marital status, place of residence, educational attainment, income, SRH, chronic disease, and eHealth literacy.

^b^Model 2 (Want) adds perceived usefulness and ease of use to the variables in Model 1.

^c^Model 3 (Adoption) includes all variables from Model 2. Adoption of eHealth services was categorized as 1 if the inpatient reported using them independently or with the help of family members and 0 otherwise.

^d^**p* < 0.05, ***p* < 0.01, *p* < 0.001.

^e^SRH: Self-rated health.

**Table S5 Logistic regression results for awareness of, want for and adoption of eHealth services**

| **Variables** |  | **Model 1^a^** |  |  | **Model 2^b^** |  |  | **Model 3^c^** |  |
| --- | --- | --- | --- | --- | --- | --- | --- | --- | --- |
|  |  | **Awareness** |  |  | **Want** |  |  | **Adoption** |  |
|  |  | **Odds ratio^d^** | **95% CI** |  | **Odds ratio** | **95% CI** |  | **Odds ratio** | **95% CI** |
| **Sex** |  |  |  |  |  |  |  |  |  |
| Female (ref: male) |  | 1.200 | [0.773, 1.862] |  | 1.287 | [0.811, 2.041] |  | 0.956 | [0.747, 1.223] |
| **Age** |  | 0.932*** | [0.915, 0.949] |  | 0.930*** | [0.910, 0.951] |  | 1.027*** | [1.014, 1.039] |
| **Marital status** |  |  |  |  |  |  |  |  |  |
| Single (ref: married) |  | 0.572 | [0.304, 1.079] |  | 0.763 | [0.364, 1.598] |  | 0.707 | [0.466, 1.074] |
| **Place of residence** |  |  |  |  |  |  |  |  |  |
| Rural (ref: urban) |  | 0.510** | [0.309, 0.841] |  | 0.378** | [0.221, 0.646] |  | 0.856 | [0.622, 1.180] |
| **Educational attainment** |  | 1.116** | [1.043, 1.195] |  | 1.085* | [1.007, 1.169] |  | 0.973 | [0.938, 1.009] |
| **Income** |  |  |  |  |  |  |  |  |  |
| Middle (ref: lowest) |  | 1.453 | [0.905, 2.334] |  | 1.341 | [0.813, 2.211] |  | 0.983 | [0.757, 1.276] |
| Highest (ref: lowest) |  | 2.296* | [1.155, 4.565] |  | 2.586* | [1.219, 5.484] |  | 0.853 | [0.549, 1.326] |
| **SRH^e^** |  |  |  |  |  |  |  |  |  |
| Fair (ref: negative) |  | 0.583* | [0.343, 0.990] |  | 0.543* | [0.322, 0.914] |  | 0.601*** | [0.450, 0.804] |
| Positive (ref: negative) |  | 0.540 | [0.282, 1.033] |  | 0.626 | [0.315, 1.241] |  | 0.418*** | [0.290, 0.601] |
| **Chronic disease** |  |  |  |  |  |  |  |  |  |
| Yes (ref：no) |  | 0.688 | [0.439, 1.103] |  | 0.802 | [0.503, 1.281] |  | 1.017 | [0.774, 1.335] |
| **EHealth literacy score** |  | 1.086*** | [1.056, 1.117] |  | 0.980 | [0.923, 1.040] |  | 1.021 | [0.993, 1.050] |
| **Perceived usefulness** |  |  |  |  | 1.457*** | [1.327, 1.600] |  | 1.091*** | [1.040, 1.145] |
| **Perceived ease of use** |  |  |  |  | 1.074 | [0.949, 1.214] |  | 0.935* | [0.884, 0.989] |

^a^Model 1 (Awareness): logistic regression model adjusted for sex, age, marital status, place of residence, educational attainment, income, SRH, chronic disease, and eHealth literacy.

^b^Model 2 (Want) adds perceived usefulness and ease of use to the variables in Model 1.

^c^Model 3 (Adoption) includes all variables from Model 2. Adoption of eHealth services was categorized as 1 if the inpatient reported using them independently and 0 otherwise.

^d^**p* < 0.05, ***p* < 0.01, *p* < 0.001.

^e^SRH: Self-rated health.

eHealth and health Survey questionnaire

Number □□□□□□

| **A Investigator Information** | | |
| --- | --- | --- |
| 1. Hospital | 1=A；2=B；3=C |  |
| 2. Investigator's Name | |  |
| 3. Investigation time | (Year/Month/Day) [eg: 20210701] |  |
| 4. Department |  |  |
| **PART1** | |  |
| **B Basic information of the patient** | |  |
| 1. Is this illness the first occurrence or a recurrence?  (1) = First occurrence [skip to question 4]; (2) = Recurrence | |  |
| 2. What was the waiting time for your most recent outpatient visit at this hospital? (in hours) | |  |
| 3. How much did you approximately spend during your most recent outpatient visit at this hospital? (in CNY) | |  |
| 4. How many days elapsed from the onset of your condition to the current hospital admission? (in days) | |  |
| 5. So far during your hospitalization, what has been the total expenditure for travel, accommodation, meals, etc.? (in CNY) | |  |
| 6. What was your reason for choosing this hospital/doctor? | 1 = Recommendation from a fellow patient;  2 = Referral;  3 = Recommendation from a friend;  4 = Introduction by another doctor;  5 = Online search;  6 = Proximity/consistent medical care |  |
| 7. What type of medical insurance do you have? | 1 = None;  2 = Urban-Rural Resident Insurance (New Rural Cooperative Medical Scheme);  3 = Provincial Direct Medical Insurance;  4 = Municipal Employee Medical Insurance |  |
| 8. Do you have any additional commercial insurance? | 1 = No;  2 = Yes |  |
| 9. Will you be able to claim reimbursement through your medical insurance this time? | 1 = No;  2 = Yes;  3 = Uncertain. |  |
| 10. What is your occupation? (Please provide the 4-digit code from the ISCO-88 classification.) | |  |
| 10-1. What is your current employment status? | 0 = Unemployed;  1 = Retired;  2 = Employed. |  |
| 11. How many years of formal education have you completed? (Do not include night school or correspondence education.) | |  |
| **The following are all inquiries about the patient’s behavior before hospitalization:** | |  |
| 12. Do you smoke? | 1 = Yes;  2 = No (If the answer is No, skip to question 17). |  |
| 13. At what age did you start smoking? | |  |
| 14. Are you currently smoking? | 1 = Yes;  2 = No (If the answer is No, skip to question 16). |  |
| 15. How many cigarettes do you smoke per day? (in cigarettes) | |  |
| 16. If you have quit smoking, how long have you been smoke-free? (in months or days) | |  |
| 17. In the past year, have you consumed beer, liquor, or other alcoholic beverages? | 1 = Yes;  2 = No (If the answer is No, skip to question 25). |  |
| 18. How often do you drink alcohol? | 1 = Almost every day;  2 = 3-4 times a week;  3 = 1-2 times a week;  4 = 1-2 times a month;  5 = Less than once a month;  6 = Don't know. |  |
| 19. In the past year, have you had beer? | 1 = Yes;  2 = No;  3 = Don't know (If the answer is No or Don't know, skip to question 21). |  |
| 20. On average, how many bottles (500 ml each) do you consume per week? | |  |
| 21. In the past year, have you had wine (including all types of fruit wine, yellow wine, rice wine, etc.)? | 1 = Yes;  2 = No;  3 = Don't know (If the answer is No or Don't know, skip to question 23). |  |
| 22. On average, how many "liang" (a Chinese measurement equivalent to 50 grams) do you consume per week? | |  |
| 23. In the past year, have you had liquor? | 1 = Yes;  2 = No;  3 = Don't know (If the answer is No or Don't know, skip to question 25). |  |
| 24. On average, how many "liang" do you consume per week? | |  |
| 25. Do you regularly engage in physical exercise or outdoor activities? | 1 = Yes;  2 = No (If the answer is No, skip to question 28). |  |
| 26. On average, how many times per week do you engage in physical exercise or outdoor activities? (in times) | |  |
| 27. On average, how long do you engage in physical exercise or outdoor activities each time? (minutes) | |  |
| 28. How long do you typically spend sitting or lying down each day while awake (e.g., watching TV, browsing the internet, chatting online, playing games, reading, working, or commuting)? (in hours) | |  |
| **C Health Status for Patient** | |  |
| Please rate your health condition: | 1 = Very Poor; 2 = Poor; 3 = Fair; 4 = Good; 5 = Very Good |  |
| **EQ-5D Scale** | |  |
| Mobility | 1 = No difficulties;  2 = Slight difficulties;  3 = Moderate difficulties;  4 = Severe difficulties;  5 = Extreme difficulties |  |
| Self-Care | 1 = No difficulties;  2 = Slight difficulties;  3 = Moderate difficulties;  4 = Severe difficulties;  5 = Extreme difficulties |  |
| Usual Activities  (e.g., Work, Study, Housework) | 1 = No difficulties;  2 = Slight difficulties;  3 = Moderate difficulties;  4 = Severe difficulties;  5 = Extreme difficulties |  |
| Pain / Discomfort | 1 = No difficulties;  2 = Slight difficulties;  3 = Moderate difficulties;  4 = Severe difficulties;  5 = Extreme difficulties |  |
| Anxiety (Nervousness, Restlessness) / Depression | 1 = No difficulties;  2 = Slight difficulties;  3 = Moderate difficulties;  4 = Severe difficulties;  5 = Extreme difficulties |  |
| **D Internet Access Basic Information** | |  |
| 1. Do you have internet access? | 1 = Yes; 2 = No |  |
| 2. Do you frequently browse short videos? | 1 = Yes; 2 = No |  |
| 3. Do you frequently post short videos? | 1 = Yes; 2 = No |  |
| 4. What electronic devices do you typically use to access the internet? (Select all that apply) | 1 = Desktop Computer; 2 = Laptop; 3 = Tablet; 4 = Smartphone |  |
| 5. Do you use a home computer to access the internet? | 1 = Never; 2 = Sometimes; 3 = Almost Daily |  |
| 6. Do you use a work computer to access the internet? | 1 = Never; 2 = Sometimes; 3 = Almost Daily |  |
| 7. Do you use a public computer to access the internet? | 1 = Never; 2 = Sometimes; 3 = Almost Daily |  |
| 8. Do you use a mobile device to access the internet? | 1 = Never; 2 = Sometimes; 3 = Almost Daily |  |
| 9. How often do you access the internet? | 1 = Once a month or less; 2 = Once a week; 3 = Several times a week; 4 = Daily; 5 = Several times a day |  |
| 10. On average, how much time do you spend on the internet each day? (in hours) | |  |

| **PART 2** | | | | | |  |
| --- | --- | --- | --- | --- | --- | --- |
| **E Patient Activation** | | | | | |  |
|  | Strongly Disagree = 1 | Disagree =2 | Agree  = 3 | Strongly Agree  = 4 | Not Applicable  = 5 |  |
| 1. Overall, I consider myself to be responsible for managing my health. | 1 | 2 | 3 | 4 | 5 |  |
| 2. Taking an active role in my healthcare is the most important factor affecting my health. | 1 | 2 | 3 | 4 | 5 |  |
| 3. I believe I can prevent or reduce health-related problems. | 1 | 2 | 3 | 4 | 5 |  |
| 4. I understand the purpose of each of my prescribed medications. | 1 | 2 | 3 | 4 | 5 |  |
| 5. I am confident in my ability to determine whether I need to see a doctor or if I can manage a health issue on my own. | 1 | 2 | 3 | 4 | 5 |  |
| 6. I believe I can express my concerns to the doctor, even if he or she does not ask. | 1 | 2 | 3 | 4 | 5 |  |
| 7. I believe I can adhere to any medical care I may need to perform at home. | 1 | 2 | 3 | 4 | 5 |  |
| 8. I am knowledgeable about my health issues and their causes. | 1 | 2 | 3 | 4 | 5 |  |
| 9. I know the treatment options available for my health issues. | 1 | 2 | 3 | 4 | 5 |  |
| 10. I have been able to maintain lifestyle changes such as proper diet or exercise. | 1 | 2 | 3 | 4 | 5 |  |
| 11. I understand how to prevent my health issues. | 1 | 2 | 3 | 4 | 5 |  |
| 12. I believe I can identify solutions when new health problems arise. | 1 | 2 | 3 | 4 | 5 |  |
| 13. I believe I can maintain lifestyle changes, such as proper diet and exercise, even during stressful times. | 1 | 2 | 3 | 4 | 5 |  |

| **F Patient Compliance** | | | | | |  |
| --- | --- | --- | --- | --- | --- | --- |
|  | Strongly Disagree=1 | Disagree=2 | Not Applicable=3 | Agree=4 | Strongly Agree=5 |  |
| 1. I am currently fully adhering to the doctor's advice. | 1 | 2 | 3 | 4 | 5 |  |
| 2. I am currently following the doctor's recommendations regarding medications. | 1 | 2 | 3 | 4 | 5 |  |
| 3. I am currently following the doctor's orders, such as remaining in bed. | 1 | 2 | 3 | 4 | 5 |  |
| 4. I have already or plan to follow the schedule recommended by the doctor for follow-up visits. | 1 | 2 | 3 | 4 | 5 |  |
| 5. I have already or plan to undergo the follow-up examinations recommended by the doctor. | 1 | 2 | 3 | 4 | 5 |  |

| **G Doctor-Patient Relationship Survey (PDRQ-9)** | | | | | |  |
| --- | --- | --- | --- | --- | --- | --- |
|  | 1=Not Applicable | 2=Slightly Applicable | 3=Applicable | 4=Quite applicable | 5=Completely applicable |  |
| 1. My doctor is helpful. | 1 | 2 | 3 | 4 | 5 |  |
| 2. My doctor gives me enough time. | 1 | 2 | 3 | 4 | 5 |  |
| 3. I trust my doctor. | 1 | 2 | 3 | 4 | 5 |  |
| 4. My doctor understands me. | 1 | 2 | 3 | 4 | 5 |  |
| 5. My doctor is eager to help me. | 1 | 2 | 3 | 4 | 5 |  |
| 6. My doctor and I agree on the issues concerning my medical symptoms. | 1 | 2 | 3 | 4 | 5 |  |
| 7. I can communicate well with my doctor. | 1 | 2 | 3 | 4 | 5 |  |
| 8. I am satisfied with the treatment provided by my doctor. | 1 | 2 | 3 | 4 | 5 |  |
| 9. I find my doctor to be approachable. | 1 | 2 | 3 | 4 | 5 |  |

| **PART 3** | | | | |
| --- | --- | --- | --- | --- |
| **H Utilization of eHealth Services** | | | | |
|  | Have you heard of it?  (0 = No; 1 = Yes) | Would you like to use this service?  (0 = No; 1 = Yes) | Have you used this service?  (0 = No; 1 = Used with family assistance; 2 = Used by myself) |  |
| 1. Online search for disease/health information |  |  |  |  |
| 2. Online search for doctor/hospital information |  |  |  |  |
| 3. Online search for doctor reviews (patients’ evaluation on doctors) |  |  |  |  |
| 4. Online consultation (text and image-based consultation) |  |  |  |  |
| 5. Communicating with other patients in groups or forums about medical conditions |  |  |  |  |
| 6. Online appointment scheduling for consultations |  |  |  |  |
| **If answered “used online appointment scheduling”, please proceed with how many times you have used this service in the past six months? (in times)** | | | |  |
| 7. Online payment for medical fees |  |  |  |  |
| 8. Online access to electronic medical records and test reports |  |  |  |  |
| 9. Online appointment scheduling for medical examinations or surgeries |  |  |  |  |
| 10. Online purchase of medications (non-supplements) |  |  |  |  |
| 11. Online management and monitoring of chronic diseases |  |  |  |  |
| 12. Online hospitalization appointment |  |  |  |  |
| 13. How did you first learn about and begin using online healthcare services? | | 1 = I was not aware that online healthcare services were available  2 = People around me (relatives, friends, etc.) informed me  3 = I learned from news reports on TV or newspapers  4 = I found out through news on my phone or computer  5 = I discovered it through short videos  6 = I was informed by a doctor or hospital staff during my visit | |  |

| **I Internet Health Information Seeking** | | | | | |  |
| --- | --- | --- | --- | --- | --- | --- |
|  | 1=Strongly Disagree | 2=Disagree | 3=Uncertain | 4=Agree | 5=Strongly Agree |  |
| 1. I will obtain new information and skills through the internet to improve my health. |  |  |  |  |  |  |
| 2. I can obtain follow-up information about new healthcare projects from the internet. |  |  |  |  |  |  |
| 3. On the internet, establishing appropriate relationships with others helps me share information on disease prevention. |  |  |  |  |  |  |
| 4. On the internet, even when I have difficulty understanding healthcare professionals, I will discuss how to perform self-monitoring with them. |  |  |  |  |  |  |
| **J eHealth Literacy (eHEALS)** | | | | | |  |
|  | 1=Strongly Disagree | 2=Disagree | 3=Uncertain | 4=Agree | 5=Strongly Agree |  |
| 1. I know how to search the internet for useful health-related information. | 1 | 2 | 3 | 4 | 5 |  |
| 2. I know how to use the internet to answer my health-related questions. | 1 | 2 | 3 | 4 | 5 |  |
| 3. I know what types of health-related information are available on the internet. | 1 | 2 | 3 | 4 | 5 |  |
| 4. I know where to find useful health-related information on the internet. | 1 | 2 | 3 | 4 | 5 |  |
| 5. I know how to use the health-related information I obtain from the internet to help myself. | 1 | 2 | 3 | 4 | 5 |  |
| 6. I possess the skills to evaluate the quality of health-related information found on the internet. | 1 | 2 | 3 | 4 | 5 |  |
| 7. I am able to distinguish between high-quality and low-quality health-related information on the internet. | 1 | 2 | 3 | 4 | 5 |  |
| 8. I am confident in making health-related decisions using information from the internet. | 1 | 2 | 3 | 4 | 5 |  |
| **K Willingness to Use eHealth services (TAM)** | | | | | |  |
|  | 1= Strongly Disagree | 2=Disagree | 3=Uncertain | 4=Agree | 5=Strongly Agree |  |
| **Perceived Usefulness** | | | | | |  |
| 1. eHealth is useful in daily health management. | 1 | 2 | 3 | 4 | 5 |  |
| 2. Using eHealth provides significant advantages in managing my health. | 1 | 2 | 3 | 4 | 5 |  |
| 3. Using eHealth benefits me. | 1 | 2 | 3 | 4 | 5 |  |
| 4. Using eHealth holds great value for my health care and medical services. | 1 | 2 | 3 | 4 | 5 |  |
| **Perceived Ease of Use** | | | | | |  |
| 5. Learning how to use eHealth is easy for me. | 1 | 2 | 3 | 4 | 5 |  |
| 6. The interaction methods I use to search for health information on the internet are clear and understandable. | 1 | 2 | 3 | 4 | 5 |  |
| 7. The use of the internet to obtain health information is flexible and easy to operate. | 1 | 2 | 3 | 4 | 5 |  |
| 8. Overall, eHealth is easy to use. | 1 | 2 | 3 | 4 | 5 |  |
| **Technology Anxiety** | | | | | |  |
| 9. I feel concerned about using eHealth. | 1 | 2 | 3 | 4 | 5 |  |
| 10. I am afraid that incorrect usage of eHealth might lead to negative consequences. | 1 | 2 | 3 | 4 | 5 |  |
| 11. I hesitate to use eHealth because of concerns about not being able to resolve errors. | 1 | 2 | 3 | 4 | 5 |  |
| 12. I find using eHealth somewhat intimidating. | 1 | 2 | 3 | 4 | 5 |  |
| **Privacy Concerns** | | | | | |  |
| 13. Using eHealth causes me to lose control over the privacy of my personal information. | 1 | 2 | 3 | 4 | 5 |  |
| 14. Using eHealth does not raise any privacy issues. | 1 | 2 | 3 | 4 | 5 |  |
| 15. Using eHealth may lead to a breach of personal privacy, as my personal information could be used without my knowledge. | 1 | 2 | 3 | 4 | 5 |  |
| 16. If I use eHealth, others may misuse my information. | 1 | 2 | 3 | 4 | 5 |  |
| **Attitude Towards Use** | | | | | |  |
| 17. Using eHealth is a good idea. | 1 | 2 | 3 | 4 | 5 |  |
| 18. Using eHealth is a wise decision and I would recommend it. | 1 | 2 | 3 | 4 | 5 |  |
| 19. I enjoy using eHealth. | 1 | 2 | 3 | 4 | 5 |  |
| **Willingness to Use** | | | | | |  |
| 20. I intend to use eHealth at some point in the future. | 1 | 2 | 3 | 4 | 5 |  |
| 21. I believe I will use eHealth in the future. | 1 | 2 | 3 | 4 | 5 |  |
| 22. I plan to use eHealth in the future, such as after I am discharged from the hospital. | 1 | 2 | 3 | 4 | 5 |  |

| **PART 4** | |  |
| --- | --- | --- |
| **L Economic Income** | |  |
| 29. How many people are there in your household? (in persons) | |  |
| 30. How do you perceive your family's economic status? | |  |
| 31. For urban residents: What is your household's average annual income level? | 1 = 118,000 CNY (approximately 9,800 CNY per month)  2 = 47,000 CNY (approximately 3,900 CNY per month)  3 = 28,000 CNY (approximately 2,350 CNY per month)  4 = 18,800 CNY (approximately 1,550 CNY per month)  5= < 18,800 CNY (approximately 1,550 CNY per month) |  |
| 32. For rural residents: What is your household's average annual income level? | 1 = 52,000 CNY (approximately 4,300 CNY per month)  2 = 21,000 CNY (approximately 1,750 CNY per month)  3 = 12,500 CNY (approximately 1,050 CNY per month)  4 = 8,400 CNY (approximately 700 CNY per month)  5= < 8,400 CNY (approximately 700 CNY per month) |  |
| **M Social Support Rating Scale (SSRS)** | |  |
| 1. How many close relationships do you have in which you can receive support and help from friends? (Select only one) | 1 = None  2 = 1-2 individuals  3 = 3-5 individuals  4 = 6 individuals or more |  |
| 2. In the past year, I have: (Select only one) | 1 = Lived away from my family and alone.  2 = Frequently moved and mostly lived with strangers.  3 = Lived with classmates, colleagues, or friends.  4 = Lived with family members. |  |
| 3. Your relationship with neighbors: (Select only one) | 1 = No mutual concern, just nodding acquaintances.  2 = May show slight concern in times of difficulty.  3 = Some neighbors are quite caring towards you.  4 = Most neighbors are caring towards you. |  |
| 4. Your relationship with colleagues/classmates: (Select only one) (**Note: If you have no colleagues/classmates, fill in 0**) | 1 = No mutual concern, just nodding acquaintances.  2 = May show slight concern in times of difficulty.  3 = Some colleagues/classmates are quite caring towards you.  4 = Most colleagues/classmates are caring towards you. |  |
| 5. Support and care received from family members | A. Spouse (or partner)  1 = None; 2 = Very little; 3 = Average; 4 = Full support |  |
|  | B. Parents  1 = None; 2 = Very little; 3 = Average; 4 = Full support |  |
|  | C. Children  1 = None; 2 = Very little; 3 = Average; 4 = Full support |  |
|  | D. Siblings  1 = None; 2 = Very little; 3 = Average; 4 = Full support |  |
|  | E. Other family members (e.g., siblings-in-law)  1 = None; 2 = Very little; 3 = Average; 4 = Full support |  |
| 6. In the past, when you encountered urgent situations, the sources of financial support and practical assistance received were:  (1) No sources.  (2) Sources from the following (select multiple options):  A. Spouse  B. Other family members  C. Relatives  D. Friends  E. Colleagues  F. Work unit  G. Official or semi-official organizations (e.g., party/group unions)  H. Non-official organizations (e.g., religious or social groups)  I. Others (how many?) | If the response is 'no sources,' then it will receive 0 points; if the response is 'the following sources,' it will receive points equal to the number of sources provided. |  |
| 7. In the past, when you encountered urgent situations, the sources of comfort and care received were:  (1) No sources.  (2) Sources from the following (select multiple options):  A. Spouse  B. Other family members  C. Friends  D. Relatives  E. Colleagues  F. Work unit  G. Official or semi-official organizations (e.g., party/group unions)  H. Non-official organizations (e.g., religious or social groups)  I. Others (how many?) | If the response is 'no sources,' then it will receive 0 points; if the response is 'the following sources,' it will receive points equal to the number of sources provided. |  |
| 8. When facing troubles, how do you express your feelings? (Select only one) | 1 = Never share with anyone.  2 = Only share with 1-2 extremely close individuals.  3 = Will share if friends ask about it.  4 = Actively express my troubles to seek support and understanding. |  |
| 9. When facing troubles, how do you seek help? (Select only one) | 1 = Rely solely on myself, do not accept help from others.  2 = Rarely ask for help from others.  3 = Sometimes ask for help from others.  4 = Often seek help from family, friends, or organizations when in difficulty. |  |
| 10. Regarding participation in activities organized by groups (e.g., party/group organizations, religious organizations, unions, student councils, etc.), you: (Select only one) | 1 = Never participate.  2 = Occasionally participate.  3 = Frequently participate.  4 = Actively participate and engage in activities. |  |
| **N Patient Information Collection Form** | |  |
| 1. Name | |  |
| 2. Sex | 1 = Male; 2 = Female |  |
| 3. Age | ____ years |  |
| 4. Ethnicity | 1 = Han; 2 = Ethnic Minority |  |
| 5. Marital Status | 1 = Married; 2 = Unmarried; 3 = Divorced; 4 = Widowed |  |
| 6. Place of Household Registration | (Province/City/District) |  |
| 7. Residential Area | 1 = Urban; 2 = Rural |  |
| 8. Admission Deposit (Prepayment, in CNY) | |  |
| 9. Diagnosis Result: (Please specify the disease name [Primary Diagnosis]) | |  |
| 10. History of Infectious Diseases | 1 = None; 2 = Hepatitis; 3 = Tuberculosis; 4 = COVID-19;  5 = Other |  |
| 11. History of Chronic Diseases | 1 = None; 2 = Hypertension; 3 = Diabetes;  4 = Coronary Heart Disease;  5 = Other (requires confirmation with the patient) |  |
| 12. History of Mental Illness | 1 = None; 2 = Mild; 3 = Severe; 4 = Severity Uncertain |  |
| 13. Family History of Genetic Diseases | 1 = None; 2 = Present |  |
| 14. History of Surgery or Trauma | 1 = None; 2 = Present |  |
| 15. Patient's Weight upon Admission | ____ kg |  |
| 16. Patient's Height upon Admission | ____ cm |  |
